# Host Resistance to *Uromyces appendiculatus* in Common Bean Genotypes

**DOI:** 10.3390/plants11050628

**Published:** 2022-02-25

**Authors:** Reda Ibrahim Omara, Said Mohamed Kamel, Sherif Mohamed El-Ganainy, Ramadan Ahmed Arafa, Yasser Sabry Mostafa, Saad Abdulrahman Alamri, Sulaiman A. Alrumman, Mohamed Hashem, Mohsen Mohamed Elsharkawy

**Affiliations:** 1 Plant Pathology Research Institute, Agricultural Research Center, Giza 12619, Egypt; redaomara43@gmail.com (R.I.O.); said_kamel88@yahoo.com (S.M.K.); sherifmsc2000@yahoo.com (S.M.E.-G.); arafa.r.a.85@gmail.com (R.A.A.); 2 Department of Arid Land Agriculture, College of Agriculture and Food Sciences, King Faisal University, Al-Ahsa 31982, Saudi Arabia; 3 Department of Biology, College of Science, King Khalid University, Abha 62529, Saudi Arabia; ysolhasa1969@hotmail.com (Y.S.M.); amri555@yahoo.com (S.A.A.); salrumman@kku.edu.sa (S.A.A.); drmhashem69@yahoo.com (M.H.); 4 Department of Botany and Microbiology, Faculty of Science, Assiut University, P.O. Box 71515, Assiut 71526, Egypt; 5 Agricultural Botany Department, Faculty of Agriculture, Kafrelsheikh University, Kafr El-Sheikh 33516, Egypt

**Keywords:** common bean, rust, disease parameters, antioxidant enzymes, reactive oxygen species, molecular markers, yield components

## Abstract

Rust, induced by the fungus *Uromyces appendiculatus*, is one of the most serious bean diseases. The involved mechanisms in rust resistance were evaluated in 10 common bean genotypes during the 2019/2020 and 2020/2021 growing seasons. The disease parameters such as final rust severity (FRS%), area under the disease progress curve (AUDPC) and disease increase rate (r-value) were lower in the resistant genotypes than in highly susceptible genotypes. Biochemical compounds such as total phenols and the activity of antioxidant enzymes such as catalase, peroxidase and polyphenol oxidase were increased in the resistant genotypes compared to susceptible genotypes. In the resistance genotypes, the levels of oxidative stress markers such as hydrogen peroxide (H_2_O_2_) and superoxide (O_2_^•−^) increased dramatically after infection. The electrolyte leakage percentage (EL%), was found to be much greater in susceptible genotypes than resistant genotypes. The resistant gene SA14, which was found in genotypes Nebraska and Calypso at 800 bp, had an adequate level of resistance to bean rust with high grain yield potential. After infection, the transcriptions levels of *1,3-D-glucanases* and *phenylalanine ammonia lyase*) were higher in the resistant genotypes than susceptible genotypes. In conclusion, the resistant genotypes successfully displayed desirable agronomic traits and promising expectations in breeding programs for improving management strategies of common bean rust disease. The resistance was mediated by antioxidant enzymes, phenolic compounds, and defense gene expressions, as well as the resistant gene SA14.

## 1. Introduction

The common bean (*Phaseolus vulgaris* L.; 2n = 2x = 22) is one of the most important leguminous vegetable crops around the world, with considerable socio-economic value for human consumption and exportation. The genus *Phaseolus* has almost 70 species, with *P. vulgaris* being the most widely cultivated [1]. For a large part of human population, dry beans constitute the primary source of vegetable protein, vitamins and minerals [2]. Rust disease, caused by the basidiomycete fungus *Uromyces appendiculatus* Pers., is a destructive disease, causing dramatic losses in crop quality and quantity across the world, and especially in Egypt [3,4]. Interestingly, humid tropical and subtropical regions are compelling conditions for the pathogen causing epidemic losses. This fungus causes yield losses from 18% to 100%, especially under the favorable conditions of a high relative humidity where epidemic losses occur [5]. In addition, there are a number of physiological races of *U. appendiculatus* that have been identified based on virulence variability.

Plants are continuously subjected to many environmental stresses, both biotic and abiotic [6]. The interactions between plants and microbes are very diverse. When a plant is subjected to a pathogen infection, it produces more salicylic acid-related enzymes, resulting in a systemic acquired resistance (SAR) response. Hydroperoxide lyase, peroxidase, and phenylalanine ammonia lyase are associated with jasmonic and salicylic acids signaling cascades [7]. Several types of defense-related genes are up-regulated in bean plants that come into contact with pathogens [8]. Plants initiate the hypersensitive response, which includes the accumulation of salicylic acid and reactive oxygen species in diseased tissues [9]. Plant growth and immunity are both influenced by reactive oxygen species (ROS). ROS have regularly been shown to increase in plants following pathogen development, and ROS have been suggested to play an essential role in plants’ defensive response [10]. Plants’ defense response, cell death, stomatal closure, and root hair growth are only a few of the numerous multifunctional roles of ROS. Free radicals such as O_2_• and OH•, as well as non-radicals such as H_2_O_2_ and O_2_•, are the primary members of the ROS family [11]. One of the first cellular reactions after effective pathogen recognition is the formation of ROS through oxygen consumption in an oxidative burst [12]. H_2_O_2_ seems to play various roles in plant defensive responses against pathogens. H_2_O_2_ has been found to decrease the development and viability of a variety of plant pathogens, possibly limiting the spread of infection. H_2_O_2_ is involved in the lignification and oxidative cross-linking of hydroxyl proline-rich proteins and other cell wall polymers, as well as other cell-wall strengthening activities. Furthermore, H_2_O_2_ has been shown to be required for the synthesis of antimicrobially active hydrophobic low-molecular-weight molecules produced in response to infection (phytoalexins). During the hypersensitive reaction, H_2_O_2_ may promote programmed plant cell death, limiting the spread of infection. It has been hypothesized that H_2_O_2_ acts as an endogenous signal in the development of SAR and induces defense genes [13].

Plants respond to stresses such as salt, drought, heavy metals, heat, and hypothermia, as well as pathogen attack, through electrolyte leakage. However, the mechanism underlying this occurrence, as well as its physiological significance, has just recently been identified. Electrolyte leakage is primarily caused by K+ efflux from plant cells [14]. One of the most important pathogen-induced impacts on plant cells is an increase in membrane permeability. The electrolyte leakage test is extensively used as a measure of plant stress tolerance and a test for stress-induced damage to plant tissues.

The identification of new genetic resources is a successful strategy to improve common bean rust resistance in different breeding programs. Since the detection of resistance genes (R-genes) is an indispensable strategy to hinder severe infections, 13 rust resistance genes have been discovered, notably Ur−1 to Ur−13 [15]. Durable resistance to common bean rust is becoming a major challenge for breeders, especially in association with new races of *U. appendiculatus* that reflect the breakdown of the single gene resistance seen in genotypes. The introduction of common bean genotypes exhibiting resistance to rust disease is an essential target of a successful breeding program around the world.

The objectives of this research were to investigate the resistance level of 10 common bean genotypes to *U. appendiculatus* under open field conditions and to explore the relation between antioxidant enzymes, ROS, oxidative stress markers and the expression of antioxidant and defense related genes in the resistant common bean genotypes.

## 2. Materials and Methods

### 2.1. Plant Materials

The current research utilized 10 genotypes of common bean, including Paulista, Samantha (Royal Sluis Ltd., Knutsford, UK), Nebraska, Bronco, Dicola, Sonata (Mascarel Co., Valencia, Spain), Fender, Karnak, Calypso and Contender (Bakker Brothers Co., Noord-Scharwoude, The Netherlands) to evaluate their resistance to bean rust disease during the 2019/2020 and 2020/2021 growing seasons.

### 2.2. Experimental Design

Common bean genotypes were screened for infection with *U. appendiculatus*. Field experiments were done in Qallin, Kafr Elsheikh Governorate (31.058959, 30.809967) during 105 days starting from the beginning of February to the middle of May (temperature 10–32 °C, relative humidity 35–56%). Seeds of all genotypes were planted in three replicates in a randomized complete block design. Each replicate has four rows, each measuring 3.5 m long and 0.6 m wide. Two seeds were put in each hole, for a total of 30 seeds sown in the row. Almost 120 seeds were sown for each genotype. The experiment was surrounded by a 1 m allay and a 1.5 m belt, which acted as a rust susceptible cultivar spreader, namely, Karnak. In addition to natural infection, the spreader was inoculated with a variety of physiological races. All agricultural practices were applied during growing seasons without fungicide application.

### 2.3. Disease Assessment

All parameters were evaluated using a 1–9 disease severity scale developed by Van Schoonhoven and Pastor-Corrales [16], with 1–3 indicating resistance (no obvious rust symptoms to slight symptoms and these genotypes can be used as a parental lines in breeding programs or/and commercial scale), 4–6 indicating intermediate resistance (distinguished symptoms and large infected area circumfluent by chlorotic halos where these germplasms are useful for disease resistance and commercial genotypes), and 7–9 indicating susceptible (occurrence of intense symptoms, epidemic losses, and plants completely dead, where these genotypes are not convenient at the commercial scale, as well as breeding programs). The tested genotypes were categorized into three groups based on infection level of rust. After *U. appendiculatus* had been fully established under field conditions, all genotypes were evaluated.

Disease severity of each particular genotype was determined by the following formula:

DS% = [Σ(n × v)/N] × 100

where:

DS = Disease severity (%).

n = Number of infected leaflets in each category.

v = Numerical value of each category (Maximum number of the scale).

N = Total number of leaflets in sample.

The disease severity (%) after reaching the complete infection of the highly susceptible check cultivar was used to calculate the final rust severity, as reported by Das et al. [17]. According to Shaner and Finney [18], the area under the disease progress curve (AUDPC) was determined for each cultivar. According to Van der Plank [19], the rate of disease increase (r-value) was determined using the following Equation:

$$r-\mathrm{value}\;=\;\frac{1}{t_{2}-t_{1}}(\log_{e}\;\frac{X_{2}}{1-X_{2}}-\log_{e}\frac{X_{1}}{1-X_{1}})$$

where:

X_1_ = Disease severity (%) at *t*_1_ date.

X_2_ = Disease severity (%) at *t*_2_ date.

*t*_2_ − *t*_1_ = Time interval in days between two observations.

### 2.4. Biochemical Assays

Ten common bean genotypes were grown in pots (25 cm) under greenhouse conditions. A randomized complete block design with three replicates was used in the experiment. The plants were fertilized as required while the pots were maintained in the greenhouse at temperatures ranging from 20 to 25 °C, a relative humidity of 70% and a photoperiod of 14 h. The urediniospore mixture was combined with talcum powder at a ratio of 1:20 (*w*/*w*) for inoculation by dusting. Urediniospores were used to inoculate 25-day-old plants. The plants were maintained in the dark for two days after being inoculated.

#### 2.4.1. Estimation of Antioxidant Enzyme Activity

After one week of infection with *U. appendiculatus*, 0.5 g of fresh bean leaf was homogenized at 0–4 °C in 3 mL of 50 mM Tris buffer (pH 7.8), with 1 mM EDTA-Na^2^ and 7.5% polyvinylpyrrolidone. The homogenates were centrifuged (12,000× *g* rpm, 20 min, 4 °C), and the total soluble enzyme activities were determined in the supernatant spectrophotometrically. According to Aebi [20], the activity of catalase (CAT) was measured. The reaction mixture containing 2 mL of sodium phosphate buffer (0.1 M, pH 6.5), 100 µL of H_2_O_2_ (0.02 M) and 50 µL of enzyme extract was used. The reduction in H_2_O_2_ was seen as a decrease in absorbance at 240 nm, and the activity was estimated using the extinction coefficient for H_2_O_2_ (0.04 mM^−1^ cm^−1^ at 240 nm). For 3 min, changes in absorbance at 240 nm were measured at 30 s intervals. The activity of the enzymes was measured in micromoles of hydrogen peroxide per gram of fresh weight per min. Malik and Singh’s [21] technique for measuring polyphenol oxidase (PPO) activity was used. Catechol solution (0.01 M, 3 mL) newly made in 0.1 M phosphate buffer (pH 6.0) was included in the reaction mixture (pH 6.0). The reaction began with the addition of 100 µL of crude enzyme extract. For 3 min, changes in absorbance at 495 nm were measured at 30 s intervals. The change in absorbance (min/g of fresh weight) was used to measure enzyme activity. According to Hammerschmidt et al. [22], peroxidase (POX) was measured in the crude enzyme extract. Two milliliters of a 0.1M sodium phosphate buffer (pH 6.0) containing 0.25% (*v/v*) guaiacol (2-methoxyphenol) and 100 mM H_2_O_2_ was used in the process. The process began with the addition of 100 µL of crude enzyme extract. For 3 min, changes in absorbance at 470 nm were measured at 30 s intervals. The extinction coefficient (26.6 mM^−1^ cm^−1^ at 470 nm) of tetra-guaiacol was used to determine the activity. The enzyme activity was measured in micromoles of tetra-guaiacol per g of fresh weight per min.

#### 2.4.2. Determination of Total Carbohydrates

Hydrolysis of dry leaf samples with NH_2_PO_4_ (for 20 min in water bath at 100 °C) was used to measure the total carbohydrates. The solution was cooled at room temperature and 90 mg of barium carbonate was added to precipitate protein. The solution was filtered into the flask, and the total volume was adjusted to 100 mL using distilled water. In this phenol solution, total carbohydrates were measured calorimetrically [23].

#### 2.4.3. Determination of Total Phenols

Total phenols were determined in methanolic extract of leaf samples using Folin-Ciocalteu reagent [24]. In a volumetric flask, the extract solution (0.1 mL) containing 1000 g of extract was mixed with 46 mL of distilled water; then, 1 mL of Folin–Ciocalteu reagent was added, and the flask was shaken. After allowing the mixture to react for 3 min, 3 mL of a 2% Na_2_CO_3_ aqueous solution was added. The absorbance was measured with a spectrophotometer at 750 nm after 2 h of incubation at 24 °C, and the total amount of phenols was calculated as micrograms of gallic acid equivalent in dry weight material.

#### 2.4.4. Evaluation of Electrolyte Leakage

The electrical conductivity of 20 discs (1 cm^2^) of bean leaves was measured to estimate the electrolyte leakage (EL%). The percentage of EL was calculated as follows: Initial conductivity/last conductivity × 100 [25].

#### 2.4.5. Evaluation of Reactive Oxygen Species (ROS)

The most prevalent free radicals of ROS are hydrogen peroxide (H_2_O_2_) and superoxide (O_2_^•^^−^), which were measured in bean leaves. Fresh leaf tissues (0.5 g) were mixed with 3 mL of K-phosphate (50 mM) buffer with a pH of 7 at 4 °C in the presence of ice. The amalgam was centrifuged for 15 min at 12,000× *g*. The top layer of the mixture (3 mL) was collected and mixed with H_2_SO_4_ (20% *v*/*v*) and TiCl4 (1%) before centrifuging for 15 min at 11,500× *g*. The absorbance of H_2_O_2_ was measured using a spectrophotometer at 410 nm and expressed as moles per gram of fresh weight [26]. The sulfanilamide technique was used to measure O_2_^•−^ production by measuring the reaction at 530 nm. The rate of O_2_^•−^ generation was measured using a NaNO_2_ reagent standard curve [27].

### 2.5. Molecular Analysis

#### 2.5.1. Genomic DNA Extraction

A hundred milligrams of bean leaf tissues were grounded in liquid N_2_ to fine powder using the coffee blender; then, the homogenized samples were transferred to 1.5 mL sterile Eppendorf tube and 500 µL of preheated CTAB extraction buffer containing 0.2% β–mercapto ethanol and mixed well before incubation at 65 °C for 30 min. Then, 500 µL of PCI (phenol/chloroform/isoamyl alcohol, 25:24:1) was added and thoroughly mixed followed by centrifugation for 15 min at 14,000× *g* rpm. The supernatant was placed into a 1.5 mL sterile Eppendorf tube with care. Isopropanol (500 µL) was pipetted to precipitate the DNA and the mixture was held on ice for 3 min before being centrifuged at 14,000× *g* rpm for 15 min. The supernatant was discarded, and DNA pellet was washed twice in 500 µL of 70% ethanol before being centrifuged for 5 min at 10,000× *g* rpm. For 20 min, the DNA pellet was air dried. The pellet was then resuspended in 75 µL of milliQ water and kept at −20 °C for further analysis.

#### 2.5.2. Polymerase Chain Reaction

To check the quality of DNA and absence of PCR inhibitors, the forward ITS5 primer (5′-GGAAGTAAAAGTCGTAACAAGG-3′) and the reverse ITS4 primer (5′-TCCTCCGCTTATTGATATGC-3′) were utilized for the amplification of the entire ITS region [28]. PCR was performed in a 25 µL reaction comprising 1 µL of the DNA extract (40 ng of total DNA), 2 mM MgCl_2_, 2.5 of 10× PCR buffer, 1.5 µL of 10 µM of each primer, 2.5 µL of 10 mM dNTPs, and 0.3 µL of 5U Taq DNA polymerase and the reaction was made up to 25 µL with nuclease-free water. Initial denaturation at 95 °C for 2 min was followed by 35 cycles of 95 °C for 30 s, 52 °C for 30 s, and 72 °C for 30 s, followed by a polymerization cycle at 72 °C for 10 min in an ESCO Swift Maxi Thermal Cycler. The presence and absence of the three resistance genes were detected using three primer pairs (Table 1).

UV light was used to observe the PCR bands, and a QIAquick Gel Extraction Kit (Cat. No. 28704) was used to remove them from the agarose gel according to the manufacturer’s instructions. Macrogen Inc. (Seoul, South Korea) sequenced the purified PCR products. Sequencing of the purified bands was performed in both directions and alignments were processed using MEGAX according to Kumar et al. [35]. Nucleotide sequence data for the selected resistance genes were submitted to the NCBI database.

#### 2.5.3. Extraction of RNA and qPCR Analysis

The RNA extraction method was carried out as previously reported at 4 days after inoculation [36]. Bean leaves were crushed into a fine powder using a mortar and pestle under liquid nitrogen. The powder was dissolved in 20 mL/g of phenol–chloroform–isoamyl alcohol and 20 mL/g of extraction buffer [36]. The mixtures were centrifuged at 12,000× *g* rpm for 10 min at 4 °C. LiCl 4 M was used to precipitate the aqueous layer and stored at 4 °C overnight. After centrifuging at 12,000× *g* rpm for 30 min at 4 °C, the pellets were washed with ethanol 70%-DEPC. The pellets were dissolved in H_2_O-DEPC and kept at 20 °C until needed. NanoDrop (Thermo Scientific, Wilmington, DE, USA) was used to measure the RNA concentrations. A High-Capacity cDNA Reverse Transcription Kit (Applied-Biosystems, Foster City, CA, USA) was used to synthesize cDNA. qPCR reactions were carried out using SYBR^®^ Green method. 2^−ΔΔCT^ was used to evaluate the qPCR results. The housekeeping gene, *Act11*, was utilized to assess the relative expression levels of the *1,3-β-D-glucanase (GLUC)* and *phenylalanine ammonia lyase* (*PAL1*). The designed primers are listed in Table 2 [37].

### 2.6. Yield Components

During the 2019/2020 and 2020/2021 growing seasons, yield components such as number of pods per plant, number of seeds per plant, and seed weight (g) per plant were measured for all genotypes.

### 2.7. Statistical Analysis

All data were statistically analyzed for each season individually. To compare treatment means, the least significant differences (*p* ≤ 0.05) was utilized. During the two growing seasons, correlation and regression coefficient “SPSS Regression Modeling” were employed to evaluate the connection between final rust severity (%) and yield components.

## 3. Results

### 3.1. Disease Assessment

Under field conditions over two seasons, the degree of resistance to bean rust was evaluated for each genotype by calculating three disease characteristics, including final rust severity (FRS%), area under the disease progress curve (AUDPC), and rate of disease growth (r-value).

#### 3.1.1. Final Rust Severity

**Figure 1:**
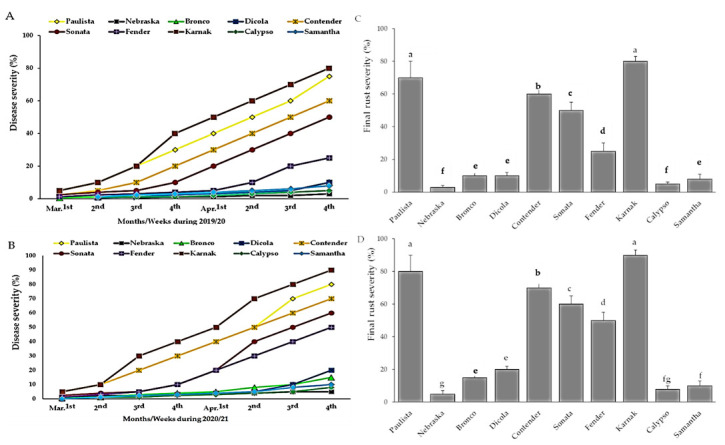
Percentage of disease severity for 10 bean genotypes during 2019/2020 (**A**) and 2020/2021 (**B**) growing seasons. Final rust severity (%) of bean rust on 10 bean genotypes, during 2019/2020 (**C**) and 2020/2021 (**D**) growing seasons. Bars represent standard errors. Different letters above columns represent significant differences.

Bean genotypes were tested under field conditions, during the 2019/2020 and 2020/2021 growing seasons. A more severe bean rust outbreak was noticed in the second season than in the first (Figure 1A,B). Although none of the assessed bean genotypes showed immunity (disease free) against *U. appendiculatus*, several genotypes showed a significant degree of rust resistance (Figure 1A,B). In the first season, the Karnak and Paulista genotypes had the highest percentage of final disease severity, followed by the extremely sensitive genotypes, Contender, Sonata, and Fender. In contrast, the Nebraska, Bronco, Dicola, Calypso and Samantha genotypes had the lowest percentage of FRS (%), indicating that they were partially resistant genotypes with an acceptable degree of adult plant resistance (Figure 1C,D). Concerning the 2020/2021 season, most of the tested genotypes had a high percentage of FRS reaching to 90% instead of the previous year’s percentage of 80% (Figure 1C,D).

#### 3.1.2. Area under the Disease Progress Curve (AUDPC)

**Figure 2:**
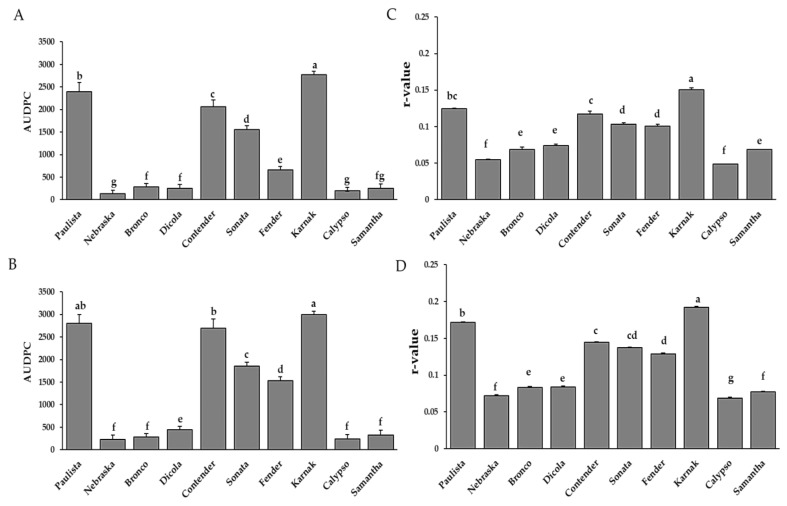
Area under disease progress curve (AUDPC) of bean rust infection on 10 bean genotypes during 2019/2020 (**A**) and 2020/2021 (**B**) growing seasons. Rate of disease increase (r-value) of bean rust infection during 2019/2020 (**C**) and 2020/2021 (**D**) growing seasons. Bars represent standard errors. Different letters above columns represent significant differences.

Different AUDPC values were obtained for the investigated bean genotypes, which were affected by minor changes in environmental conditions during each growing season. However, compared to the first season, the field conditions in the second season were apparently more favorable for disease onset and development. Thus, the highest AUDPC estimations were observed in the highly susceptible genotypes, during the second season (Figure 2A,B). Similar results may be seen in the first season, but at lower values (Figure 2A,B). The bean genotypes may be divided into two major categories based on the data collected and AUDPC estimations. The first group included bean genotypes with the lowest AUDPC estimates (less than 500) such as Nebraska, Bronco, Dicola, Calypso and Samantha during the 2019/2020 and 2020/2021 seasons (Figure 2A,B). These genotypes were, therefore, designated as partially resistant (PR) ones, since they displayed the highest and most acceptable levels of adult plant resistance (APR) or field resistance, under the stress of bean rust disease, throughout the two seasons of the study. The second group included the highly susceptible genotypes (more than 500) such as Karnak, Paulista, Contender, Sonata and Fender. These genotypes showed the lowest levels of APR and the highest estimates of AUDPC in both seasons (Figure 2A,B). Therefore, the results indicated that this group of genotypes might be categorized as the fast-rusting group.

#### 3.1.3. Rate of Disease Increase (R-Value)

In 2019/2020 and 2020/2021 seasons, r-value was calculated using the obtained data of rust severity (%) from the time of rust appearance to the early dough stage. The genotypes could be divided into two main groups (Figure 2C,D). The first group included five genotypes that had a high disease incidence (more than 1). These genotypes were Karnak, Paulista, Contender, Sonata and Fender. The second group included five genotypes (Neraska, Bronco, Dicola, Calypso and Samantha), which exhibited low r-value rates (less than 1).

### 3.2. Total Carbohydrates and Phenols

**Figure 3:**
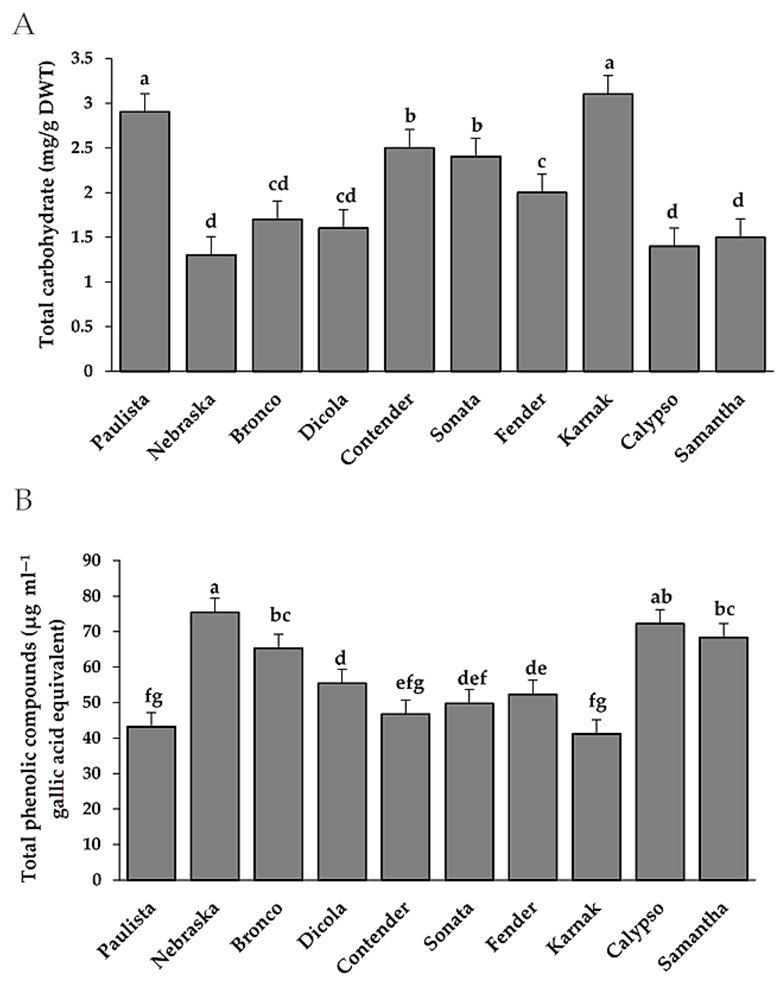
Total carbohydrates (**A**) and total phenolic compounds (**B**) in different bean genotypes infected with *U. appendiculatus*. Bars represent standard errors in the data set. Different letters above columns represent significant differences.

Total carbohydrates and total phenols were determined in 10 bean genotypes in order to explain their levels in relation to infection with *U. appendiculatus* (Figure 3). The bean susceptible genotypes such as Karnak, Paulista, Contender, Sonata and Fender exhibited high total carbohydrates levels (Figure 3). On the contrary, resistant genotypes such as Nebraska, Bronco, Dicola, Calypso and Samantha exhibited low total carbohydrates levels.

### 3.3. Estimation of Antioxidant Enzymes

**Figure 4:**
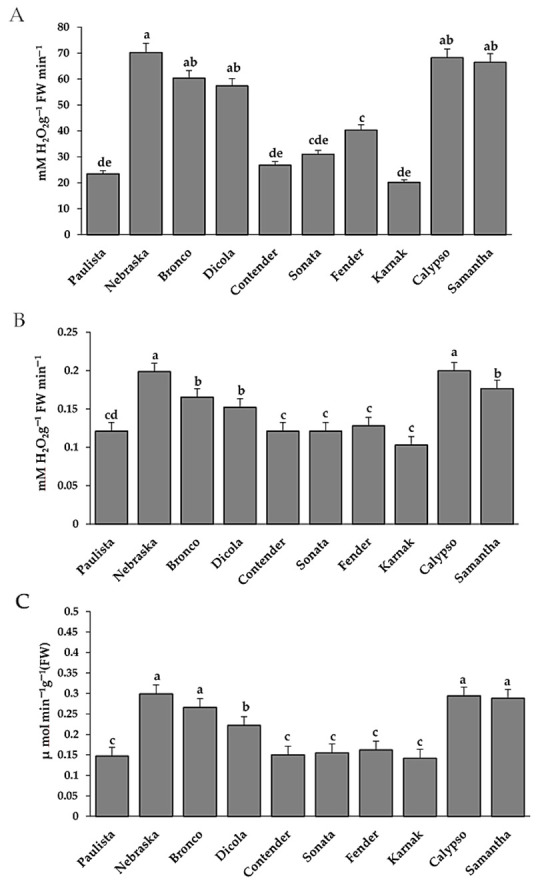
Catalase (**A**), peroxidase (**B**) and polyphenol oxidase (**C**) activities in different bean genotypes infected with *U. appendiculatus.* Bars represent standard errors in the data set. Different letters above columns represent significant differences.

The activity of the antioxidant enzymes peroxidase (POX), polyphenol oxidase (PPO) and catalase and (CAT), which are used to indicate stress in bean plants, was higher in resistant bean genotypes than in susceptible genotypes (Figure 4). The antioxidant enzymes POX, PPO, and CAT were significantly increased in resistant bean genotypes. Nebraska and Calypso produced the greatest POX, PPO, and CAT activities compared to the other genotypes.

### 3.4. Estimation of Hydrogen Peroxide and Superoxide Anion and Electrolyte Leakage

**Figure 5:**
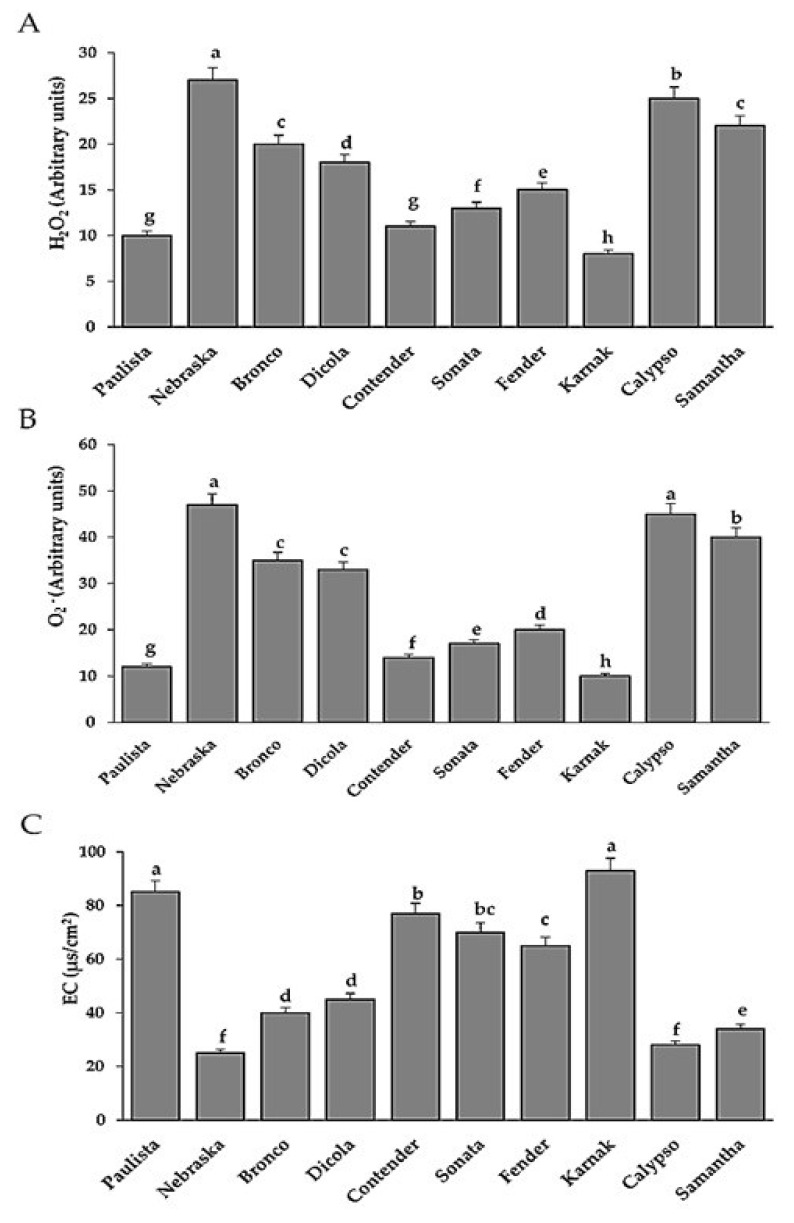
Hydrogen peroxide (**A**), superoxide anion (**B**) and Electrolyte leakage (**C**) in different bean genotypes infected leaves by *U. appendiculatus*. Bars represent standard errors in the data set. Different letters above columns represent significant differences.

In resistant bean genotypes, the accumulation of H_2_O_2_ and O_2_^•−^ was considerably higher than in susceptible bean genotypes. The H_2_O_2_ and O_2_^•−^ levels increased substantially in the resistant genotypes Nebraska, Bronco, Dicola, Calypso, and Samantha (Figure 5). The susceptible genotypes such as Karnak, Paulista, Contender, Sonata, and Fender showed lower quantities of H_2_O_2_ and O_2_^•−^. The electrolyte leakage percentage (EL%), a measure of cell membrane permeability, was found to be much greater in susceptible bean genotypes than in resistant genotypes (Figure 5).

### 3.5. Molecular Analysis

**Figure 6:**
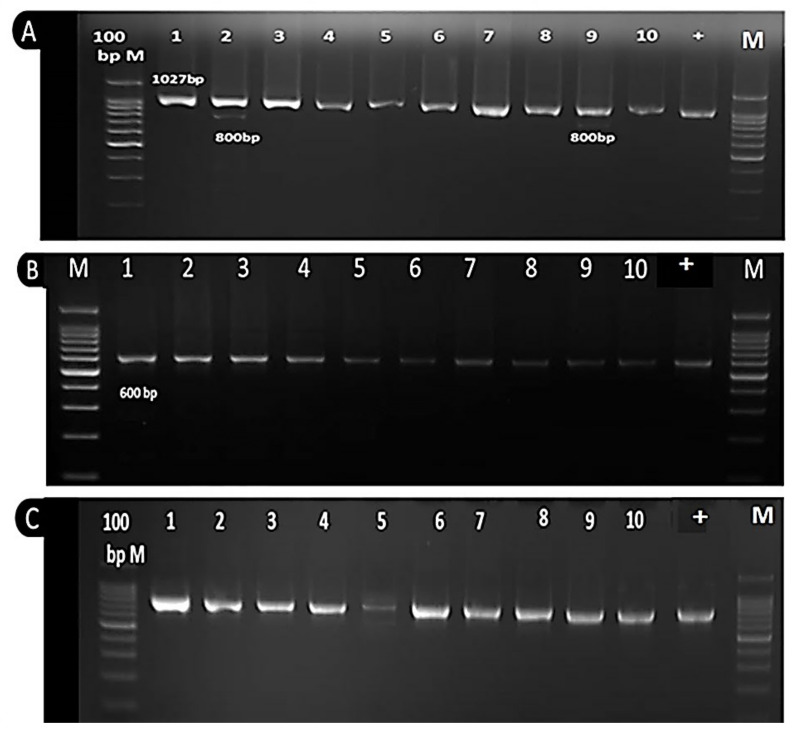
Molecular profiling of 10 common bean genotypes using primers SA14F/R (**A**), SF10F/R (**B**), and ITS4 and ITS5 (**C**). Lanes 1–10 represent the common bean genotypes: Paulista, Nebraska, Bronco, Dicola, Contender, Sonata, Fender, Karnak, Calypso and Samantha, respectively. Lane M is 100 bp ladder.

A total of three sequences amplified region (SCAR) markers (SK14, SA14 and SF10) were examined for banding patterns in 10 selected common bean genotypes. The amplification profile of SCAR markers SA14F/R and SF10F/R were presented in Figure 6A,B, however no bands were detected with the SK14F/R marker. Interestingly, the obtained results revealed that the susceptibility gene was homozygous in all tested genotypes at 1079bp, whereas the resistant gene of SA14F/R was heterozygous in two genotypes only (Nebraska and Calypso) at 800 bp (Figure 6A). Moreover, the PCR amplicon of SF10F/R was observed in all tested genotypes as homozygous at 600bp (Figure 6B). Moreover, the obtained findings demonstrated that the resistance gene Ur-3 was absent in all tested genotypes, whereas Ur-4 was present in eight genotypes as a homozygous form and heterozygous in two genotypes. Additionally, ITS4 and ITS5 were used as a positive control (Figure 6C).

To obtain more accurate results, the PCR amplicons of two common bean genes along with 10 genotypes were sequenced with accession numbers MT512396, MT512397 and MT512398 (Supplementary Table S1). The obtained results demonstrated that three forms released two forms (resistance and susceptible) related to the SA14 primer associated with the Ur-4 gene, whereas the third form was linked to the marker SF10 which was tightly associated with rust resistance in the Ouro Negro (Ur-ON) cultivar.

Interestingly, there were differences between the two forms of the SA14 marker in the number and position of the single-nucleotide polymorphisms (SNPs) on the genome of common bean.

### 3.6. Transcription of Defense-Related Genes

**Figure 7:**
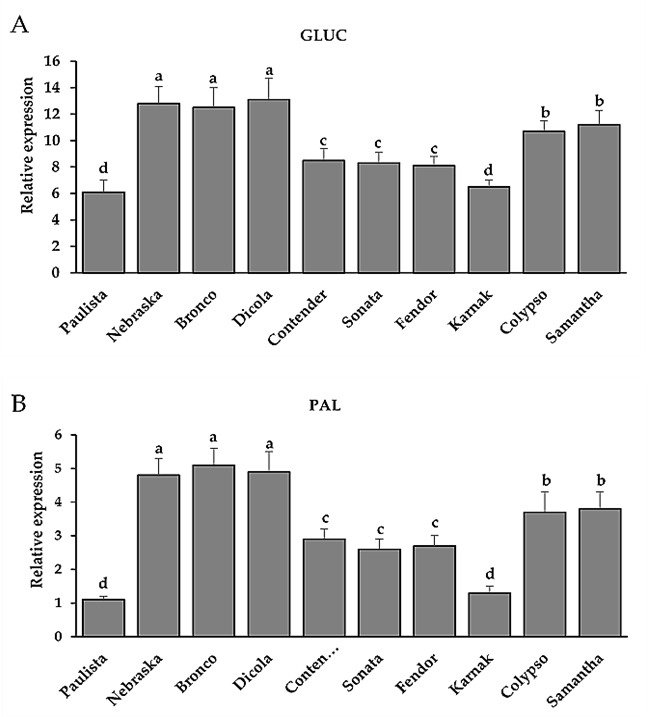
Effect of inoculation of different bean genotypes with *U. appendiculatus* on transcript levels of the defense-associated genes *1,3-D-glucanases* (**A**) and *phenylalanine ammonia lyase* (**B**) at 4 days after inoculation based on the reference gene (*Actin*). Transcript levels were estimated using real-time quantitative reverse transcription polymerase chain reaction (qRT-PCR). Bars represent standard errors in the data set. Different letters above columns represent significant.

Four days after infection with *U. appendiculatus*, real-time PCR (qRT-PCR) technology was used to measure transcript levels of antioxidant genes coding for *1,3-D-glucanases (GLUC)* and *phenylalanine ammonia lyase* (*PAL*) in the leaves of bean seedlings. The *GLUC* and *PAL* transcript levels in the leaves of the bean genotypes Nebraska, Bronco, and Dicola were significantly up-regulated in infected seedlings compared to other genotypes (Figure 7). There were no significant changes in the expression of defense-related gene transcripts between the three genotypes, Nebraska, Bronco and Dicola. This increase varied from 2 to 13-fold depending on the bean genotype and the gene analyzed. The highest defense gene expressions were recorded in Dicola for *GLUC* (13-fold) and Bronco for *PAL* (5-fold).

### 3.7. Correlation Analysis between SA14 Gene, Antioxidant Enzymes, Hydrogen Peroxide, Superoxide Anion, Electrolyte Leakage and Gene Expressions of GLUC and PA

Pearson correlation matrix was determined between SA14 gene, antioxidant enzymes, hydrogen peroxide and superoxide anion, electrolyte leakage and gene expressions *GLUC* and *PAL* (Table 3). Except for the relationships between electrolyte leakage, which were negatively relevant, all factors in Table 3 were positive related.

### 3.8. Assessment of Yield Parameters

**Figure 8:**
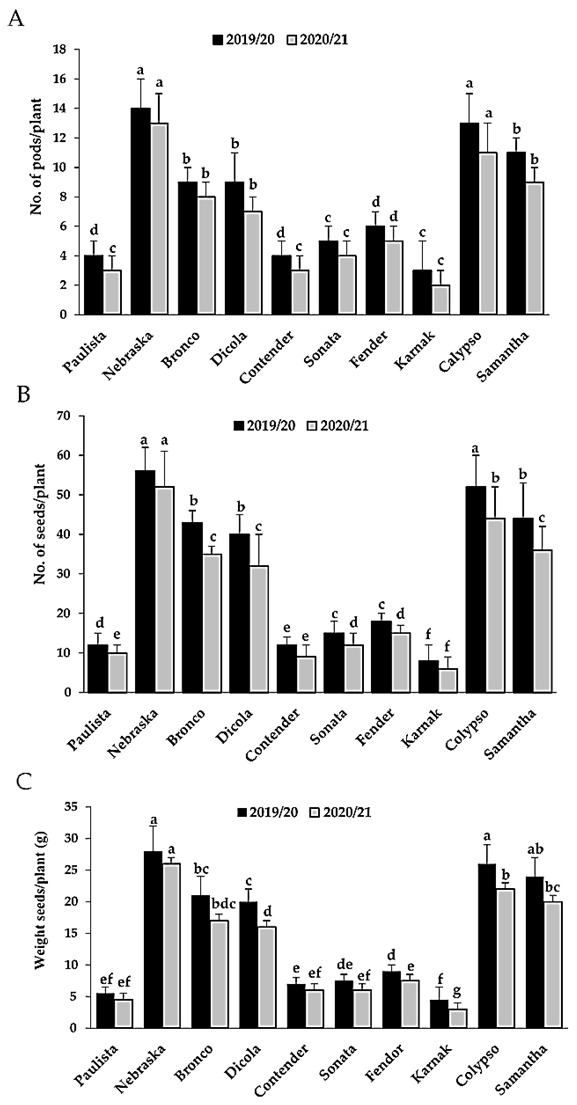
Effect of bean rust infection on number of pods per plant (**A**), number of seeds per plant (**B**) and weight seeds (g) per plant (**C**) on 10 genotypes during 2019/2020 and 2020/2021 growing seasons. Bars represent standard errors. Different letters above columns represent significant differences.

The findings confirmed that the yield assessment parameters including number of pods per plant, number of seeds per plant and weight seeds (g) per plant, during the 2019/2020 and 2020/2021 growing seasons were significantly different among the 10 tested genotypes (Figure 8). The estimated yield components of the different genotypes under the present study were determined. Actually, there was a quantitative relationship between yield components and disease parameters of rust disease. The highest number of pods per plant, number of seeds per plant and weight seeds (g) per plant were recorded with the highly resistant or partial resistant genotypes; Neraska, Bronco, Dicola, Calypso and Samantha. In contrast, the lowest yield components were recorded in highly susceptible or fast-rusting genotypes; Karnak, Paulista, Contender, Sonata and Fender.

### 3.9. Correlation Analysis between Final Rust Severity and Yield Components

**Figure 9:**
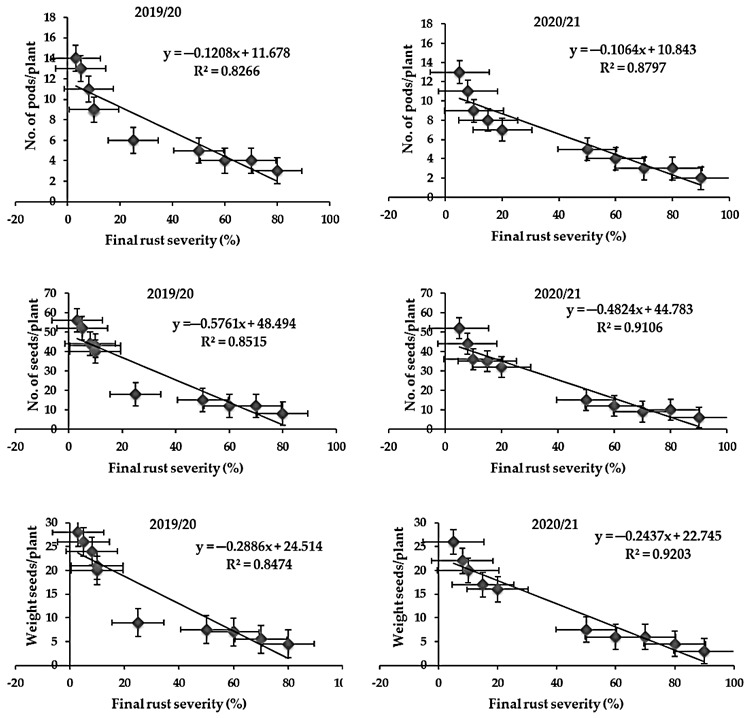
Correlation coefficient between FRS (%) and each of number of pods per plant, number of seeds per plant and seeds weight (g) per plant during 2019/2020 and 2020/2021 growing seasons.

Relationships between FRS (%) and yield components (no. of pods/plant, no. of seeds/plant and weight seeds (g)/plant) were determined through correlation coefficient analysis, during 2019/2020 and 2020/2021 growing seasons (Figure 9). In season 2019/2020, there were strong positive relations between the FRS (%) under study and each of no. of pods/plant, no. of seeds/plant and seeds weight (g)/plant, where estimates of R^2^ were 0.8266, 0.8515 and 0.8474, respectively (Figure 9). On the other hand, in the second season (2020/2021), the relations between FRS and yield components were stronger, where estimates of R^2^ were 0.8797, 0.9106 and 0.9203 of no. of pods/plant, no. of seeds/plant and seeds weight (g)/plant, respectively (Figure 9). It can be concluded that the relation between FRS (%) and yield components was more stable in 2020/2021 season.

## 4. Discussion

Bean rust (*U. appendiculatus*) is a serious disease affecting common bean production worldwide [15]. Biochemical and molecular investigations of cultivated germplasm are important for breeding programs in order to gain new information and knowledge about the existing genotypes under open field conditions. Since this domain is extremely significant in common bean breeding programs, this study aimed to identify novel genetic sources of rust disease resistance in common bean genotypes. The resistant genotypes play a crucial role for rust management strategies and combating the permanent threats all over the world.

Under field conditions, adult plant resistance (APR) was experimentally measured and characterized using three disease parameters; final rust severity (FRS%), area under disease progress curve and rate of disease increase as well as yield components. During the two years of the study, FRS (%) recorded the lowest values on the APR genotypes—Nebraska, Bronco, Dicola, Calypso, and Samantha. In contrast, the highly susceptible genotypes (Karnak, Paulista, Contender, Sonata and Fender) exhibited the lowest levels of APR to bean rust infection and were severely rusted and showed the highest estimates of FRS (%), in both seasons. Consequently, it was concluded that Neraska, Bronco, Dicola, Calypso and Samantha, had the potential to decrease the amount of bean rust infection in both seasons of the study. The obtained results are in agreement with EL-Awady and Hamed [38] who mentioned that 22 cultivated genotypes were evaluated naturally against rust pathogen, where the Samantha genotype was resistant; however, four genotypes Concessa, Hort. 440, Coby and Hana were immune. The AUDPC and r-value, as good estimators of APR, were widely applied and used by many investigators. All of them mentioned that AUDPC was a reliable and more convenient estimator of APR than any other parameter. They represent both the amount of rust infection and the rate at which the disease or pathogen has increased during an epidemic period [4,39,40]. A slight decrement in the level of bean rust severity, expressed as lower estimates of FRS (%), AUDPC and r-value in the first season (2019/20) less than those in the second season (2020/21), could be due to the slight differences in environmental conditions between the two years under study. Another reason is the diversity or variation in the prevalent bean rust virulent pathotypes from one year to another within the pathogen populations, where more than 300 races or pathotypes of *U. appendiculatus* were identified indicating the broad range of variability of this pathogen [41]. Similarly, Arunga et al. [42] showed that the physiological races of the *U. appendiculatus* population have changed and new races were recorded at the beginning of the 21st century. Unfortunately, there is little information regarding the virulence diversity of *U. appendiculatus* races in Egypt and this is a great challenge in breeding programs. Therefore, enhancement and gene pyramiding of bean genotypes with multi-genes of resistance to rust is an important strategy to manage the disease. It is clear that some local common bean genotypes have an adequate level of field resistance or APR to bean rust and many of them served in agriculture for many years, showing high levels of APR during their vast cultivation under field conditions. This result is considered the most motivating output for breeding programs and crop improvement strategies to prophesy by the performance of new common bean genotypes and introgression of resistance genes [43].

The results confirmed that chemical compounds such as total phenols and the enzyme activities such as catalase, peroxidase and polyphenol oxidase were increased in the resistant genotypes. On the contrary, total carbohydrates were increased in the susceptible genotypes compared to the resistant genotypes. The fast production of phenolics in the cell wall enable plant defense against disease. Many of the plant’s defense mechanisms against a fungal disease are associated with pathways that control sugar levels in the cell and maintain the plant’s energy balance [44]. This could explain the reason for the increase in total phenols and carbohydrates in resistant genotypes. In cucurbit genotypes, up-regulation of the catalase (CAT) enzyme plays an important role in increasing reactive oxygen species (ROS), to secure cucurbit plants from pathogen attack because CAT is one of the antioxidant enzymes that plays an important role in controlling plant diseases [45,46,47]. According to Neamat et al. [48], increased levels of CAT prevents an increase in cytosolic hydrogen peroxide that creates toxic conditions, leading to oxidative stress and preventing the spread of pathogens. It acts as a secondary signal for the expression of defense genes and activation of systemic acquired resistance. Peroxidase (POX) is the first enzyme to show changes in its activity under environmental stress [49]. Moreover, the changes in this antioxidant enzyme are known to be directly involved in the activation of plant defense responses [50]. POX is known to accelerate the final polymerization stage of lignin production and is associated with systemically protected tissues’ enhanced capacity to lignify [51].

Pathogen attack and other stresses caused alterations in plant membrane permeability and electrolyte leakage (EL), which was typically accompanied by an increase of ROS and sometimes resulted in programmed cell death [14,46,52,53]. The EL test is extensively used as a measure of plant stress tolerance and a test for stress-induced damage to plant tissues. Bean rust disease was enhanced EL in bean plants in the current research. Rust fungi are obligate parasites that rely on their host cells for nutrition. Although the cell membrane in the resistant genotype was unaffected by pathogen attack, the cell membrane in the susceptible genotype lost its components due to pathogen inoculation [45]. The resistant genotype exhibited a substantial reduction in membrane permeability compared to susceptible genotypes.

Undoubtedly, molecular markers are considered one of the best approaches for genetic variability analysis and resistance genes detection at nucleic acids level [54]. Various types of molecular marker strategies have been generated through substantive changes of the biological system and creation of new theories on the molecular levels. Regarding common bean, different molecular markers have been utilized for genetic diversity, characterization, identification of resistance locus and fine mapping like random amplified polymorphic DNA, microsatellite, sequence characterized amplified region and single nucleotide polymorphism [55]. In the present study, genetic diversity and polymorphism were investigated among 10 common bean distinct genotypes using SCAR markers. The SCAR SF10 marker is associated with rust resistance gene (Ur-ON) in common bean cultivar Ouro Negro which is one of the best genetic resources for *U. appendiculatus* in Brazil [31]. Since this marker was amplified in all tested genotypes, these genotypes can be used as parental lines for breeding programs against rust resistance. The outcome of the present research exhibited high correlation between phenotypic dataset and molecular marker analysis.

*U. appendiculatus* infection caused a substantial elevation in the expression levels of *GLUC* and *PAL* genes in bean seedlings, indicating that the pathogen infection promoted systemic accumulations of antioxidant enzymes *GLUC* and *PAL* [56]. *GLUC* and *PAL* are two important enzymes in the phenylpropanoid pathway, which contribute to the formation of phytoalexins [57]. *GLUC* is an enzyme that hydrolyzes the cell wall components known as β-glucan that are found in the cell walls of fungi. The findings of this research clearly demonstrated that *GLUC* and *PAL* expression is not limited to the location of infection, but quickly extended to all leaves. As a result, this occurrence indicates the presence of a signal that spreads in a systemic way in the plant. In the current research, endogenous ROS, such as hydrogen peroxide (H_2_O_2_) and superoxide anion (O_2_^•−^), rose early after infection in the resistant genotypes. Therefore, ROS play an essential role in plant defensive responses [10]. The increase in H_2_O_2_ makes it difficult for microbes to penetrate plants. H_2_O_2_ is the key chemical generated by plants during an oxidative burst. Invading pathogens are inhibited or killed by high ROS levels, which also promote programmed cell death in infected plant cells (HR) and oxidative crosslinking of cell wall components (penetration resistance) [12]. Therefore, plant defense responses are linked to antioxidant activity and H_2_O_2_ accumulation in bean resistance to rust pathogen. The possible reason is that ROS trigger pathogen restriction, which results in the death of host plant cells at inoculation sites, and that function as signaling molecules, inducing antioxidant and pathogenesis-related defensive responses in surrounding plant cells. After being inoculated with *U. appendiculatus*, resistant common bean genotypes promoted the formation of ROS, which were strongly linked to the resistance gene (SA14 gene), as well as POX, PPO, CAT, GLUC and PAL to limit the spread of the invading pathogen. The results showed that the resistance package, which included ROS, R-gene, and antioxidant enzymes, had a beneficial impact on the common bean’s immune system against rust disease through several mechanisms.

Successful breeding program aim to combine high and adequate level of resistance to bean rust with high grain yield potential in advanced genotypes. The results relevant to number of pods per plant, number of seeds per plant and seeds weight per plant showed significant differences between the tested genotypes as affected by the level of disease severity. This result was confirmed by correlation coefficient analysis where, there were strong positive relationships between the FRS (%) and yield components. Similar results were previously obtained by Souza et al. [5] who reported that the yield losses of rust disease in bean genotypes ranged from 18% to 100%, especially under favorable conditions with high relative humidity where epidemic damages occurred.

## 5. Conclusions

The potential of new genotypes with resistance genes could be utilized as donor parents in breeding programs. This study evaluated the response of 10 common bean genotypes to rust infection caused by *U. appendiculatus*. The populations of common bean exhibited beneficial genetic resources for breeding programs, genetics, and genomics research. The results showed that the antioxidant enzymes, levels of oxidative stress markers and defense genes are involved in rust resistance. The present research adds to plant breeders’ arsenal of genetic resources for developing novel resistant and/or tolerant cultivars resistant to common bean rust disease. The resistant genotypes that possess multiple traits associated with resistance are beneficial since plants are exposed to different types of diseases that participate in yield losses. These observations also will lead to a reduction in fungicide treatments and a boost in legume crop yield productivity.

## Figures and Tables

**Table 1:** List of the sequence characterized amplified region (SCAR) markers used in the PCR reaction.

No.	Gene	Oligo Name	Oligo Seq.	Accession Number	Size (bp)	Reference
1	SK14	SK14F	CCC GCT ACA CAC CAA TAC CTG	XM_007159311	620	[29,30]
		SK14R	CCC GCT ACA CTT GAT AAA ATG TTA G			
2	SA14	SA14F	CTA TCT GCC ATT ATC AAC TCA AAC	XM_007140094	1079/800	[30,31,32]
		SA14R	GTG CTG GGA AAC ATT ACC TAT T			
3	SF10	SF10F	GGA AGC TTG GTG AGC AAG GA	XM_007151078	620	[31,33,34]
		SF10R	GGA AGC TTG GCT ATG ATG GT			

**Table 2:** Common bean sequences used for primer design for RT-PCR analysis.

Primer Name	Forward Primer (5′–3′)	Reverse Primer (5′–3′)	Accession Number	Product Size (pb)
*GLUC*	GCTGTAAGGGCTCAAGGCCTC	CCAAGTACACACGTGCGTTGTC	X53129	427
*PAL*	AAGCCATGTCCAAAGTGCTG	GAGTTCTCCGTTGCCACCT	M11939	240
*ACTIN*	CACCGAGGCACCGCTTAATC	CGGCCACTAGCGTAAAGGGAA	AB067722	126

**Table 3:** Pearson correlation matrix between SA14 gene, antioxidant enzymes, Hydrogen peroxide and superoxide anion, electrolyte leakage and gene expressions.

	SA14	CAT	POX	PPO	H_2_O_2_	O_2_^•−^	GLUC	PAL	EL
SA14	1	0.667 *	0.667 *	0.667 *	0.816 **	0.816 **	0.816 **	0.816 **	−1.000 **
CAT		1	0.583	1.000 **	0.816 **	0.816 **	0.816 **	0.816 **	−0.667 *
POX			1	0.583	0.816 **	0.816 **	0.816 **	0.816 **	−0.667 *
PPO				1	0.816 **	0.816 **	0.816 **	0.816 **	−0.667 *
H_2_O_2_					1	1.000 **	1.000 **	1.000 **	−0.816 **
O_2_^•−^						1	1.000 **	1.000 **	−0.816 **
GLUC							1	1.000 **	−0.816 **
PAL								1	−0.816 **
EL									1

* Correlation is significant at the 0.05 level. ** Correlation is significant at the 0.01 level.

## Data Availability

Not applicable.

## Supplementary Materials

The following supporting information can be downloaded at: https://www.mdpi.com/article/10.3390/plants11050628/s1, Table S1: Sequencing of two common bean genes where Ba2_B refer to the resistant group of the first gene (SA14F/R marker), whereas Ba10_B refer to the susceptible group of the same gene. BF10_FR refers to the second gene (SF10F/R marker) of common bean.
