# Supplementary material for: Host Resistance to Uromyces appendiculatus in Common Bean Genotypes
Source: Plants (Basel). 2022 Feb 25;11(5):628. doi: 10.3390/plants11050628 (PMC8912588; doi:10.3390/plants11050628)
Supplement: Supplementary file 1 [file plants-11-00628-s001.zip › plants-1575388-supplementary.pdf]

Supplementary Table 1. Sequencing of two common bean genes where Ba2_B refer to the resistant group of the first gene (SA14F/R marker), whereas Ba10_B refer to the susceptible group of the same gene. BF10_FR refers to the second gene (SF10F/R marker) of common bean.

Marker	Accession number	Sequencing
SA14F/R (Ba2_B)	MT512396	GGATTTTTCCAGTGTAAACCAAATTAGGACATAGCCGAAAAA<br>TGATAGTCAAATTGAAAAGAAGAGACACTATGGAAGAGGAG<br>ACTAATTGGTACAGCTCTAATATGATGTTTTTAAAGAGAAAA<br>ATAGATGCAAGAGATATTGAAACTGAGTACACAATATTTCAG<br>TGCGTTTTATATAGAGTTAATGAAACAAATACAAATAAATAT<br>ACGAAATAATATTCCTATTCATATGATATGATTAGACCTATCA<br>TTACAAAGATCTTGATTAAATATATTACAAAGTGGACTTTAAG<br>CCTAACTCAACCCCATAAAACCGGCTCATAGAGTTGAGATTT<br>GCACCCACTTATATACAATGAAAGACTCTAATCTCTAGTCGAT<br>GTGGGATCTCCAACACACCCCCCTCACGCCGAGACTGCCAAC<br>TCGTGCGTGAGACTATATATTATGGGTGATCCGATAGCGGGTG<br>GCACGATAGGCCCAACACAAATACTCGCTAGGATAGACTCGA<br>AATGGCTCTGATACCATATTACAAAGTGGACTTTAAGCCTAA<br>CTCAACCCCATAAAAAATAAGAAAGCAAAAATCATAAAATA<br>AAAAATATCATCTAGAATATTCTAAAATATTCTAAGATATATA<br>AAAAAATATCATGAGATATTTGTCAGATTTTCTAACAGCTACT<br>CCCAATGAGGCACTGGAATATGTACCAGTATATTTAGCATCC<br>ACACATAAAGCTTAGACTTACTGGTCATGCTGCTTCTCAACCC<br>AGACTTCCATCCATGAGGAAATTTGATTCTCTGCATAGATAAG<br>ATCATGTGGAAGATTATCAAGTACCTACACAAAGTAAAGTAA<br>TTGTTGTTAAATATCTAAATAACAATAACAATAATAAAAAAA<br>ATTCACCATTAATGAGAAGAAAACACCAGCAAAAGAAGATT<br>TCTAGTCTGCAACATTGAATACCA
SA14F/R marker (Ba10_B)	MT512397	CATGGATTTTTCCAGTGTAAACCAAATTAGGACATAGCCGAA<br>AAATGATAGTCAAATTGAAAAGAAGAGACACTATGGAAGAG<br>GAGACTAATTGGTACAGCTCTAATATGATGTTTTTAAAGAGAA<br>AAATAGATGCAAGAGATATTGAAACTGAGTACACAATATTTC<br>AGTGCGTTTTATATAGAGTTAATGAAACAAATACAAATAAAT<br>ATACGAAATAATATTCCTATTCATATGATATGATTAGACCTAT<br>CATTACAAAGATCTTGATTAAATATATTACAAAGTGGACTTTA<br>AGCCTAACTCAACCCCATAAAACCGGCTCATAGAGTTGAGAT<br>TTGCACCCACTTATATACAATGAAAGACTCTAATCTCTAGTCG<br>ATGTGGGATCTCCAACACACCCCCCTCACGCCGAGACTGCCA<br>ACTCGTGCGTGAGACTATATATTATGGGTGATCCGATAGCGG<br>GTGGCACGATAGGCCCAACACAAATACTCGCTAGGATAGACT<br>CGAAATGGCTCTGATACCATATTACAAAGTGGACTTTAAGCCT<br>AACTCAACCCCATAAAAAATAAGAAAGCAAAAATCATAAAA<br>TAAAAAATATCATCTAGAATATTCTAAAATATTCTAAGATATA<br>TAAAAAAATATCATGAGATATTTGTCAGATTTTCTAACAGCTA<br>CTCCCAATGAGGCACTGGAATATGTACCAGTATATTTAGCATC<br>CACACATAAAGCTTAGACTTACTGGTCATGCTGCTTCTCAACC

		CAGACTTCCATCCATGAGGAGATTTGATTCTCTGCATAGATAAGATCATGTGGAAGATTATCAAGTACCTACACAAAGTAAAGTAATTGTTGTTATATATCTAAATAACAATAACAC
SF10F/R marker (BF10_FR)	MT512398	GTTCAAAACAATTTAAAATATATCTTAATAGTTTTTTCCTGTGCTCCACCAACCACTCATCTAAGATCAAATCCAGTTCTTTGGCAGTTTCTTTCATGGCTTTCACTCCCAAATTCATCAATCTCAGAACAGGAACAGCATCAGCCACTGTGCACACCCCCTGTTAAGAAACTCTGATAACTGACACACTTTATTGAATAATAGAAAAGCTAATATATAGCTGATTACATAAACTGACTATCCTAAAACATAGCTAGAAAATAGGAACTAATAAAGACTAAATCAAACTGAATCCAAGTGCTATGCTAACAGCCCTATAAAATAGGACATTTATTCTAACAAACTAACCAAGTCAGAATTAACCATATCATTTCCTCCCTTAAACTAGACAGTATACTAGTTTAACTTAGGTGCATCACATACTCCAAGTTTCTTCCTAAGATGCTGAAATGTCTTCAACTTGAGAGGCTTAGTGAAAATGTCTGCTATCTGTTGTTCAGTGCCACAAAACTTCAGTTGTACAGCTCCTTCATTCACCATATCACGTAGGAAATGGAACCTTACATCAATGTGCTTACTCTTTCCGTGCATCACAGGGTTTTTGGATA
